# Seed Biochemical Analysis Based Profiling of Diverse Wheat Genetic Resource from Pakistan

**DOI:** 10.3389/fpls.2017.01276

**Published:** 2017-07-20

**Authors:** Anam Khalid, Amjad Hameed

**Affiliations:** ^1^Nuclear Institute for Agriculture and Biology Faisalabad, Pakistan; ^2^Pakistan Institute of Engineering and Applied Sciences Islamabad, Pakistan

**Keywords:** enzymatic antioxidants, non-enzymatic antioxidants, wheat, nutritional quality

## Abstract

Wheat is the major nutrient source worldwide. In Pakistan, it has a crucial place in agriculture as well as in national economy. For seed biochemical compositional analysis, wheat germplasm (77 genotypes) was collected from different agro-climatic zones of Pakistan. Significant variation (*p* < 0.05) was observed for tested parameters among tested genotypes. Highest activity of ascorbate peroxidase (APX) was detected in Pavon (1,426.67 Units/g s. wt.), catalase (CAT) in Pasban-90 (633.33 Units/g s. wt.), peroxidase (POD) in IQBAL-2000 (42,579.6 Units/g s. wt.), and superoxide dismutase (SOD) in Manthar-2003 (278.93 Units/g s. wt.). Whereas, maximum activity of alpha amylase was found in SH-2002 (292.70 mg/g s. wt.), esterase in Dharabi 2011 (987.80 μM/min/g s. wt.), and protease in NR-234 (11,183.33 Units/g s. wt.) and highest total oxidant status (TOS) was detected in Faisalabad-2008 (390.0 μM/g s. wt.), malondialdehyde (MDA) content in Margalla-99 (679.23 μM/g s. wt.), total phenolic content (TPC) in Bhakkar-2000 (25,383.33 μM/g s. wt.), and ascorbic acid (AsA) content in SH-2002 (713.0 μg/g s. wt.). Maximum total soluble sugar was found in Saleem-2000 (29.86 mg/g s. wt.), reducing sugars in Punjab-96 (12.68 mg/g s. wt.), non-reducing sugars in Saleem-2000 (27.33 mg/g s. wt.). However, highest albumins was identified in TC-4928 (352.89 mg/g s. wt.) and globulins in MEXI PAK (252.67 mg/g s. wt.), salt soluble proteins in Faisalabad-2008 (162.44 mg/g s. wt.), and total soluble proteins in Punjab-96 (487.33 mg/g s. wt.) indicating good quality of wheat genotypes as well as good nutritional status. Genotypes which have been ranked high in respective parameter can be employed in breeding to enhance the nutritional quality of wheat.

## Introduction

Wheat is the major nutrient source worldwide as it provides >20% of proteins as well as calories to the large number of individuals living predominantly in developing countries (Braun et al., 2010; Goutam et al., 2013). Production of wheat is focused primarily in China, Russia, Turkey, Indian, Ukraine, Australia, United States, and Pakistan, accounting for above 80% of the global wheat production (Ikhtiar and Alam, 2007; Siddiqui and Naz, 2009; Sun et al., 2014). Pakistan is the eighth leading producer of wheat, accounting for almost 3.17% of the wheat output in the world from 3.72% of the area under wheat cultivation (Shuaib et al., 2010). In Pakistan, wheat is a principal food grain which has a crucial place in agriculture as well as economy (Khan et al., 2007; Shuaib et al., 2010). Besides the fundamental nutrients such as carbohydrates and proteins, wheat is an excellent source of wide range of phytochemicals, providing supplementary benefits regarding health. It is a very fine source of antioxidants and remarkable anti oxidative activity has been observed in wheat and wheat based foodstuffs (Okarter et al., 2010; Angioloni and Collar, 2011).

In order to retain normal biological functions and activities, an appropriate balance is required between antioxidants and free radicals. Free radicals are naturally generated within the body to accomplish several daily metabolic actions of cell but sometimes the extent of these free radicals increases because of intrinsic and extrinsic aspects causing discrepancy in the prevailing antioxidants. This escalation harms the molecules of cells mostly the DNA, protein, and lipids of membrane that give rise to marked up occurrence of degenerative ailments for example brain abnormalities, cancer, arthritis, cataract, and cardiac syndromes. Body of humans encloses various endogenous substances which scavenge free radicals; however, a significant portion is contributed through intake also. Whole grains, fruits, and vegetables possess enormous kinds of phytochemicals and most of them are liable for the antioxidative action of these foodstuffs (Narwal et al., 2014).

Enzymatic antioxidants like superoxide dismutase (SOD), glutathione reductase (GR), and ascorbate peroxidase (APX), catalase (CAT) and peroxidase (POD) are capable of removing hydrogen peroxide, taking away free radicals and intermediates of oxygen in mitochondria along with chloroplast (Lee et al., 2007). The non-enzymatic antioxidants comprise two common classes i.e., antioxidants associated with membrane which is lipid soluble such as alpha tocopherol and beta carotene and water soluble reducers like ascorbate, glutathione, and phenolics (Jaleel et al., 2009).

The recent interest in the benefits of whole cereal grain with respect to health has encouraged breeders to rise the wheat antioxidants concentration (Sun et al., 2014). The aim of this study was to examine the difference in the content of antioxidant among ancient cultivars, land races of wild relatives of domesticated crop, and advance as well as segregating lines of wheat. Efforts were made to discover the antioxidant activity of wheat grains, Hence, detailed analysis was conducted to explore the biochemical composition of wheat grains using diverse wheat genotypes available in Pakistan, which will offer a chance to discover the available wheat genotypes with superior nutritional quality.

## Materials and methods

Wheat germplasm of 77 varieties harvested during the year 2015 were collected from various centers within each agro-climatic zone of Pakistan for testing the antioxidant activity (Table 1). The samples were stored in labeled glass bottle to ensure preserve integrity. The analysis was carried out at Marker Assisted Breeding Lab—1 Plant Breeding and Genetics Division, Nuclear Institute for Agriculture and Biology (NIAB), Faisalabad, Pakistan.

**Table 1:** Wheat varieties used in this study.

**Sr. no**.	**Variety**	**Year of release**	**Source**	**Genotype pedigree**	**Sr. no**.	**Variety**	**Year of release**	**Source**	**Genotype pedigree**
1	PAVON	1978	AARI, Faisalabad	VCM//CNO/7C/3/KAL/BB	16	INQULAB 91	1991	AARI, Faisalabad	WL 711/CROW ‘S’
2	PERWAZ	1994	AARI, Faisalabad	V5648/(SIB)PARULA[1604]	17	SEHAR 2006	2006	AARI, Faisalabad	CHILL/2^*^STAR/4/BOW//BUC/PVN/3/2^*^VEE#10
3	MEXI-PAK	1965	AARI, Faisalabad	PJ/GB55 or PJ62/GB55	18	CHAKWAL 50	2008	BARI, Chakwal	ATTILA/3/HUITLE/CARCOMUN(CARC)//CHEN/(CHTO)CHORLITO/4/ATILA
4	LU-26	1976	UAF	BLUESILVER/KHUSHAL-69	19	BARS 2009	2009	BARI, Chakwal	“PFAU/SERI//BOW”
5	IQBAL-2000	2000	AARI, Faisalabad	BURGUS/SORT 12-13//KAL/BB/3/PAK 81	20	DHARABI 2011	2011	BARI, Chakwal	HXL7573/2^*^BAU//PASTOR
6	AUQAB-2000	2000	AARI, Faisalabad	CROW‘S’/NAC//BOW'S	21	FAISALABAD 2008	2008	AARI, Faisalabad	PBW65/2^*^Pastor
7	UFAQ-2002	2002	AARI, Faisalabad	V.84133/V83150	22	AARI 2011	2011	AARI, Faisalabad	SH-88/90A-204//MH97
8	GA-2002	2002	BARI, Chakwal	DWL5023/SNB//SNB	23	MILLAT 2011	2011	AARI, Faisalabad	CHENAB2000/INQ-91
9	BHAKKAR-2000	2000	AZRI, Bhakkar	P102/PIMA//F3.71/TRM/3/PVN	24	PUNJAB 2011	2011	AARI, Faisalabad	AMSEL/ATTILA//INQ-91/PEW‘S’
10	SH-2002	2002	AARI, Faisalabad	INQALAB-91/FINK‘S’	25	GALAXY 2013	2013	AARI, Faisalabad	–
11	AS-2002	2002	AARI, Faisalabad	KHP/D31708//CM74A370/3/CNO79/4/RL6043/4^*^NAC or KHP/D31708//CM74A370/3/CIANO79/4/RL6043/^*^4NAC	26	V-8203/Ujala 16	2016	AARI, Faisalabad	4777^*^2/5/CIANO67/8150//TOBARI66/CIANO67/4/NOROESTE66/3/12300//LERMAROJO64/8156/6/(SIB)TRIFON
12	MANTHAR-2003	2003	RARI, Bahawalpur	KAUZ//ALTAR84/AOS	27	BAKHTAWAR-1993	1993	NIFA, Peshawar	KAUZ ‘S’
13	FAREED-2006	2006	RARI, Bahawalpur	PT‘S’/3/TOB/LFN//BB/4/BB/HD-832-5//ON/5/G-V/ALD‘S’//HPO	28	TATARA 1996	1996	NIFA, Peshawar	JUP/ALD‘S’//KLT‘S’
14	SHAFAQ 2006	2006	AARI, Faisalabad	LU26/HD21790/2^*^INQALAB 91	29	FAKHR-E-SARIAD	1998	NIFA, Peshawar	PFAU‘S’/SERI//BOW‘S’
15	MAIRAJ 2008	2008	RARI, Bahawalpur	PIAMONTESINTA/3/TOBARI66/LILIFEN//BLUEBIRD/4/BLUEBIRD/HD-8325//OLESEN/5/GAVILAN,MEX/(SIB)ALONDRA//(SIB)HOOPOESPARROW/INIA//V.7394/WL711/13/BAUS	30	TAKBEER 2000	2000	NIFA, Peshawar	ATTILLA
31	LASANI 2008	2008	AARI, Faisalabad	PFAU/SERI//BOW	45	NIFA LALMA 2012	2012	NIFA, Peshawar	–
32	JAUHAR-78	1979	NIA, Tando jam	(M)NAYAB; PENJAMO-62/GABO-55//GABO-66/3/TEZANOS-PINTOS-PRECOZ/NAINARI-60/4/NF-600-RADS	46	WATAN-94	1994	AARI, Faisalabad	LU-26/HD-2179
33	SINDH-81	1982	NIA, Tando jam	NORTENO-67/MEXIPAK	47	MARVI-2000	2002	NIA, Tando jam	CMH77A917/PKV1600//RL6010/6^*^SKA
34	SARSABZ	1986	NIA, Tando jam	PITIC62/FROND//MEXIPAK/3/PITIC-62/MAZOE-79-75-76	48	ABADGAR-93	1996	NIA, Tando jam	CIANO-F67/NOROESTE-F66/3/C273//NP875/PITIC-62/4/PRATAP
35	SOGHAT-90	1991	NIA, Tando Jam	PAVON MUTANT-3	49	C-228	1941	AARI, Faisalabad	HARD FED/9D
36	KIRAN-95	1996	NIA, Tando Jam	CROW(SIB)/W-711	50	C-591	1934	AARI, Faisalabad	T9/8D or T9 X 8A
37	BARANI-83	1983	AARI, Faisalabad	BB/GLL/3/GTO/7C//BB/CNO67	51	02006	–	AARI, Faisalabad	–
38	BHITTAI 2004	2004	NIA, Tando jam	VEERY/TRAP//SOGHAT-90	52	02005	–	AARI, Faisalabad	–
39	SASSUI-2006	2006	NIA, Tando jam	CHIL /ALD//PVN/Yecora-70	53	02156	–	AARI, Faisalabad	SH-88/WEAVER
40	KHIRMAN 2006	2006	NIA, Tando jam	ULC/PVN//TAN/3/BUC	54	KOHISTAN	1997	AARI, Faisalabad	V1562//CHIROCA(SIB)/(SIB)HORK/3/KUFRAI/4/CARPINTERO(SIB)/(SIB)BLUEJAY
41	NIA AMBER	2010	NIA, Tando jam	VEE//5‘S’/SARA//SOGHAT90	55	9021	–	UAF	FENGMAI10/MO120//110-75/3/ZHONGKANGAI-2/4/04835/5/882-182
42	NIA SUNHARI	2010	NIA, Tando jam	Cham4//URES/BOW ‘S’	56	TC-4928	–	UAF	–
43	NIA SUNDAR	2011	NIA, Tando jam	sarsabz/sunco^*^2	57	6544-6	–	UAF	–
44	BENAZIR-12	2012	NIA, Tando jam	CHEN/AEGILOPSSQUARROSA(TAUS)//BCN/3/VEE#7/	58	9244	–	AARI, Faisalabad	–
59	NARC-2011	2011	NARC, Islamabad		69	NR-234	–	NARC, Islamabad	–
60	ZARDANA	1993	ARI, Quetta	CIANO67/8156//TOBARI66/CIANO67/4/NOROESTEF66/3/12300//LERMA-ROJO-64-A/8156/5/PAVON-76	70	NR-241	–	NARC, Islamabad	–
61	RASKOH-2005	2005	ARI, Quetta	Kauz/Yaco//Kauz	71	MARGALLA-99	1999	NARC, Islamabad	OPATA/BOW‘S’
62	MEHRAN-89	1991	–	KVZ/BUHO//KAL/BB	72	SITTA	1991	CIMMYT	–
63	PUNJAB-90	1990	AARI, Faisalabad	–	73	NESSER	1990	CIMMYT	–
64	SA-75	1975	AARI, Faisalabad	NAI60/CB151/S949/3/MEXIPAK	74	SALEEM 2000	2001	CCRI, Pirsabak	CHAM6//KITE/PAPAGO-86
65	PASBAN-90	1991	AARI, Faisalabad	–	75	SULEMAN96	1996	CCRI, Pirsabak	F674/BUNTING//SISKIN/3/VEERY-7
66	NIFA-BATHOOR	2008	NIFA, Peshawar	–	76	PUNJAB-96	1996	AARI, Faisalabad	SA42^*^2/4/CC/INIA//BB/3INIA/HD832 or SA42^*^2/3/CC/INIA4//BB/4/INIA/HD832
67	PARI 73	1973	AARI, Faisalabad	CNO67//SN64/KLRE/3/8156	77	WL-711	1978	AARI, Faisalabad	S308/CHRIS//KAL
68	S-24	–	UAF	–	–	–	–	–	–

*AARI, Ayub Agriculture Research Institute; ARI, Agriculture Research Institute; RARI, Regional Agriculture Research Institute; NARC, National Agriculture Research Center; CCRI, Cereal Crop Research Institute; NIA, Nuclear Institute for Agriculture; NIFA, Nuclear Institute for Food and Agriculture; UAF, University of Agriculture Faisalabad*.

### Evaluation of antioxidant enzyme activities

#### Germplasm extraction

Wheat grains (0.2 g) were dry grinded and extracted in 2 ml (50 mM) potassium phosphate buffer (pH 7.4) or in buffer specified in method. Samples were centrifuged at 14,462 × g for 10 min at 4°C. The supernatant was separated and used for the determination of different enzymatic and non-enzymatic activities. All the data was taken in triplicate.

### Enzymatic antioxidants

#### Ascorbate peroxidase (APX) activity

For the estimation of APX activity, seeds were homogenized in a medium composed of 50 mM potassium phosphate buffer (pH 7.0). APX activity was measured using the method of (Dixit et al., 2001). Assay buffer was prepared by mixing 200 mM potassium phosphate buffer (pH 7.0), 10 mM ascorbic acid and 0.5 M EDTA. For measurement of APX, the activity assay solution contained assay buffer made up of 10 mM ascorbic acid, 0.5 M EDTA and 200 mM potassium phosphate buffer, H_2_O_2_ (1 ml), and supernatant 50 μl. The oxidation rate of ascorbic acid was estimated by following the decrease in absorbance at 290 nm after every 30 s (Chen and Asada, 1989).

#### Catalase (CAT) activity

For the estimation of catalase activity, seeds were homogenized in a medium composed of 50 mM potassium phosphate buffer (pH 7.0) and 1 mM dithiothreitol (DTT). Catalase (CAT) was estimated using the method described by Beers and Sizer (1952). For measurement of CAT activity, the assay solution contained 50 mM phosphate buffer (pH 7.0), 59 mM H_2_O_2_, and 0.1 ml enzyme extract. The decrease in absorbance of the reaction solution at 240 nm was recorded after every 20 s. An absorbance change of 0.01 min^−1^ was defined as 1 U of CAT activity. Enzyme activity was expressed on seed weight basis.

#### Peroxidase (POD) activity

For the estimation of POD, seeds were homogenized in a medium composed of 50 mM potassium phosphate buffer (pH 7.0), 0.1 M EDTA, and 1 mM DTT. Activity of peroxidase (POD) was measured using the method of Chance and Maehly (1955) with some modification. For measurement of POD activity, the assay solution contained distilled water (545 μl), 200 mM phosphate buffer (pH 7.0), 200 mM guaiacol, 400 mMH_2_O_2_, and 15 μl enzyme extract. The reaction was initiated after adding the enzyme extract. The increase in absorbance of the reaction solution at 470 nm was recorded after every 20 s. One unit of POD activity was defined as an absorbance change of 0.01 min^−1^. Enzyme activity was expressed on seed weight basis.

#### Superoxide dismutase (SOD) activity

For the estimation of SOD activity, seeds were homogenized in a medium composed of 50 mM potassium phosphate buffer (pH 7.0), 0.1 mM EDTA, and 1 mM dithiothreitol (DTT) as described by Dixit et al. (2001). The activity of SOD was assayed by measuring its ability to inhibit the photochemical reduction of nitroblue tetrazolium (NBT) following the method of (Giannopolitis and Ries, 1977). One unit of SOD activity was defined as the amount of enzyme that caused 50% inhibition of photochemical reduction of NBT.

### Non-enzymatic antioxidants

#### Total phenolic content (TPC)

A micro colorimetric method as described by Ainsworth and Gillespie (2007) was applied with some modifications for total phenolics content, in which Folin-Ciocalteau (F-C) reagent was used. For analysis, 0.5 g seed samples were homogenized in 500 μl ice cold 95% methanol using an ice cold mortar and pestle. The samples were then incubated at room temperature for 48 h in the dark. The samples were then subjected to centrifugation at 14,462 × g for 5 min at room temperature. The supernatant was removed and used for TPC measurement. A 100 μl of supernatant was mixed with 100 μl of 10% (v/v) F-C reagent, vortex thoroughly, and then 800 μl of 700 mM Na_2_CO_3_ was added. Samples were then incubated at room temperature for 1 h. Blank corrected absorbance of samples was measured at 765 nm. A standard curve was prepared using different concentration of gallic acid and a linear regression equation was calculated. Phenolic content (gallic acid equivalents) of samples was determined using linear regression equation.

#### Ascorbic acid

For ascorbic acid determination, the 2,6-dichloroindophenol (DCIP) method described by Hameed et al. (2005), which measures reduced ascorbic acid only was used. Briefly, each molecule of vitamin C converts a molecule of DCIP into a molecule of DCIPH_2_, and that conversion can be monitored as a decrease in the absorbance at 520 nm. A standard curve was prepared using a series of known ascorbic acid concentrations. A simple linear regression equation was calculated to find the ascorbate concentration in unknown samples.

### Hydrolytic enzymes

#### Esterases activity

The α-esterases and β-esterases were determined according to the method of Van Asperen (1962) using α-naphthyl acetate and β-naphthyl acetate as substrates, respectively. The reaction mixture consisted of substrate solution [30 mM α or β-naphthyl acetate, 1% acetone, and 0.04 M phosphate buffer (pH 7)] and enzyme extract. The mixture was incubated for exactly 15 min at 27°C in dark and then 1 ml of staining solution (1% Fast blue BB and 5% SDS mixed in ratio of 2:5) was added and incubated for 20 min at 27°C in dark. Amount of α- and β-naphthol produced was measured by recording the absorbance at 590 nm. Using standard curve, enzyme activity was α or β naphthol produced in μM min^−1^ per g seed weight.

#### Protease activity

For estimation of protease activity, seed samples were extracted in 50 mM potassium phosphate buffer pH 7.8. Protease activity was dictated by the casein digestion assay described by Drapeau (1974). By this method one unit is that amount of enzyme, which releases acid soluble fragments equivalent to 0.001 A280 per min at 37°C and pH 7.8. Enzyme activity was expressed on seed weight basis.

#### Alpha amylase activity

The alpha amylase activity of wheat seeds was determined by the modified method as reported by the (Varavinit et al., 2002).

### Other biochemical parameters

#### Total oxidant status (TOS)

Total oxidant status (TOS) was determined by using (Erel, 2005) formulated method which is based on the oxidation of ferrous ion to ferric ion by oxidants present in the sample in an acidic medium and the measurement of ferric ion by xylenol orange (Harma et al., 2005). The assay mixture contained reagent R_1_, reagent R_2_ and sample extract. After 5 min. the absorption was measured at 560 nm by using spectrophotometer. A standard curve was prepared using hydrogen peroxide. The results were expressed in μM H_2_O_2_ equivalent per L.

#### Malondialdehyde (MDA) content

The level of lipid peroxidation in the seed flour was measured in terms of malondialdehyde (MDA, a product of lipid peroxidation) content determined by the thiobarbituric acid (TBA) reaction using method of Heath and Packer (1968) with minor modifications as described by Dhindsa et al. (1981). A 0.25 g seed sample was homogenized in 0.1% TCA. The homogenate was centrifuged at 14,462 × g for 5 min. To 1 m aliquot of the supernatant 20% TCA containing 0.05% TBA were added. The mixture was heated at 95°C for 30 min and then quickly cooled in an ice-bath. After centrifuging at 14,462 × g for 10 min, the absorbance of the supernatant at 532 nm was read and the value for the non-specific absorption at 600 nm was subtracted. The MDA content was calculated by using extinction coefficient of 155 mM^−1^ cm^−1^.

#### Reducing sugars (sugar content)

Level of reducing sugars in seeds was determined by the dinitrosalicylic acid method (Miller, 1959). Total sugar contents were estimated by the phenol–sulphuric acid reagent method (Dubois et al., 1951). Non-reducing sugars were calculated by the difference.

### Differential protein estimation

#### Protein content

For protein estimation, seeds were homogenized in a medium composed of 50 mM potassium phosphate buffer (pH 7.0). Estimation of quantitative protein was executed by previously described method (Bradford, 1976). For protein estimation in samples, 5 μl of supernatant and 0.1 N NaCl were mixed with 1.0 ml of Bradford dye and the mixture was allowed to stand for 5 min to form a protein dye complex. Absorbance was calculated at 595 nm by using spectrophotometer.

#### Albumins

The first protein extract was obtained by adding 10 ml buffer A (0.01 M Tris-HCl pH 7.5) in 0.02 g seed flour (obtained through dry grinding the seed samples). After 2 h stirring at room temperature, the mixture was centrifuged at 4,000 g for 10 min and the supernatant corresponding to the albumins fraction was recovered. The pellet was then washed with 10 ml of buffer A, centrifuged, and the supernatant corresponding to the albumins fraction was recovered. The pellet was then washed with 10 ml of buffer A, centrifuged, and the supernatant pooled with the previous one. This fraction was albumin extraction (Bradford, 1976).

#### Globulins

The pellet was mixed with 10 ml of buffer B (0.01 M Tris-HCl pH 7.5, and 1 M NaCl) and stirred for 2 h before centrifuging at 4,000 g for 10 min, also twice. This fraction was the globulin extraction (Bradford, 1976).

#### Salt soluble proteins

Seeds samples were dry grinded using pestle and mortar and 0.02 g seed flour was added in 2.5 ml of 0.15 M potassium phosphate (pH 7.5), 2.5 ml 5 mM DTT, and stirred 0.5 h at room temperature before centrifuging at 2,000 g for 5 min, three times extracted as before. The protein fraction obtained in the recovered supernatant was referred to as salt soluble protein (Bradford, 1976).

### Statistical analysis

All the data was reported as mean ± SD. Descriptive statistics was applied to analyse and organize the resulting data. Data were analyzed using two-way ANOVA with replications. Significance of data was tested by analysis of variance and Tukey (HSD) Test at *p* < 0.05 and where applicable at *p* < 0.01 using XL-STAT software. Data was also subjected to principal component analysis using computer software Microsoft Excel along with XLSTAT Version 2012.1.02, Copyright Addinsoft 1995-2012 (http://www.xlstat.com).

## Results

### Enzymatic antioxidants

#### Super oxide dismutase (SOD) activity

Genotypes were divided in three categories (low, medium, and high) based on comparative values of different studied parameters (Table 1).

For SOD activity in wheat seeds, significant variation was found among wheat genotypes and it was possible to categorize them in low, medium, and high classes. Thirty-eight genotypes were grouped in high category for SOD, in which the values ranged from 171 to 279 Units/g s. wt. Mean values for studied parameters are presented as Figure S1. Among these genotypes, highest SOD activity was found in Manthar-2003 (278.93 Units/g s. wt.). Sixteen genotypes with SOD activity ranging from 151 to 170 Units/g s. wt. were grouped as intermediate class. Twenty-three genotypes were grouped in third category that is genotypes having comparatively low SOD activity. In this class, genotypes having SOD activity <150 Units/g s. wt. were grouped. Among these genotypes having comparatively low SOD activity, the lowest value (76.17 Units/g s. wt.) was observed in Punjab-2011.

#### Catalase (CAT) activity

Genotypes were divided in three categories based on comparative values of different studied parameters (Table 2). For catalase activity in wheat seeds, significant variation was also found among wheat genotypes and it was possible to categorize them in low, medium, and high classes. Four genotypes were grouped in high category for CAT, in which the values ranged from 368 to 634 Units/g s. wt. Mean values for catalase are presented as Figure S2. Among these genotypes, highest CAT activity was found in Pasban-90 (633.33 Units/g s. wt.). Thirty-eight genotypes with CAT activity ranging from 101 to 367 Units/g s. wt. were grouped as intermediate class. Thirty-five genotypes were grouped in third category that is genotypes having comparatively low CAT activity. In this class, genotypes having CAT activity <100 Units/g s. wt. were grouped. Among these genotypes having comparatively low APX activity, the lowest value was observed in Nesser (46.67 Units/g s. wt.).

**Table 2:** Scale for categorization of wheat genotypes in low, medium, and high value for different biochemical parameters.

**Parameters**	**Range of values**					
	**Low**	**Genotypes**	**Medium**	**Genotypes**	**High**	**Genotypes**
SOD (Units/g s. wt.)	≤150	Punjab-2011, Sassui-2006, Nia Sunhari, Sarsabz, Fakhar-e- Sarhad, WL-711 Kohistan, NR-234, NARC-2011, PAVON	151–170	Dharabi 2011, 2006, Punjab-96, 6544-6, Pasban-90, Suleman, Benazir-12, 9021, Marvi, Fareed-2006	171–279	Zardana, PERWAZ, LU-26, Bakhtawar-1993, Mairaj-2008, Nifa Lalma 2012, Nia Amber, Saleem-2000, 2156, Takbeer-2000
CAT (Units/g s. wt.)	≤100	Nesser, Watan-94, Bhittai-2004, Sassui-2006, Khirman2006, Nia Amber, 2005, 2006 Bhakkar-2000, Sindh-81	101–367	TC-4928, Abadghar-93, NR-421, IQBAL-2000, Nifa Lalma 2012, MEXI PAK Kohistan, Dharabi 2011, GA-2002, Shafaq-2006	368–634	WL-711, S-24, SA-75, Pasban-90
APX (Units/g s. wt.)	≤400	NARC-2011, 9021, Benazir-12, Marvi, BARS-2009, 2006, AARI-2011, Punjab-90, Abadghar-93 Kohistan	401–999	Pasban-90, Barani-83, Jauhar-78, Nia Sundar, SH-2002, Sassui-2006, Takbeer-2000, Zardana, Kiran- 95, Raskoh-2005	1,000–1,427	Pari-73, WL-711, MEXI PAK, PERWAZ, PAVON
POD (Units/g s. wt.)	≤9,000	Punjab-90, PAVON, SA-75, PERWAZ, Pasban-90, Pari-73, WL-711, Punjab-2011, Nifa- Bathoor, Benazir-12	9,001–19,999	Margalla-99, Zardana, Mehran-89, Faisalabad-2008 Sarsabz, Nia Sunhari, TC-4928, Takbeer-2000, Lasani-2008, 2005	20,000–42,580	6544-6, Tatara 1996, Chakwal-50 Bhakkar-2000, AARI-2011, UFAQ 2002, IQBAL-2000
TPC (μM/g s. wt.)	≤9,700	WL-711, Pasban-90, Punjab-90, Punjab-96, Pari-73, Nifa- Bathoor, S-24, Saleem-2000, SA-75, Barani-83	9,701–20,232	Lasani-2008, 2006, Sitta, Khirman2006, Kiran- 95, Jauhar-78, Raskoh-2005, Fakhar-e- Sarhad, Mairaj-2008, Mehran-89	20,233–25,383	PAVON, Inqulab-91, Fareed-2006 Nia Sunhari, Bhittai-2004, 2005, UFAQ 2002, Faisalabad-2008, AS-2002, 9244
AsA (μg/g s. wt.)	≤640	S-24, Pasban-90, Punjab-96, WL-711, SA-75, Punjab-90, Pari-73, Nifa- Bathoor, Millat-2011, AARI-2011	641–681	Mehran-89, Bhittai-2004 Abadghar-93, Nia Sundar, Nia Sunhari, NR-421, Nifa Lalma 2012, Margalla-99, Saleem-2000, 9021	682–713	Sehar-2006, Inqulab-91, Zardana, 2156, Punjab-2011, Barani-83, NR-234, Khirman2006, Watan-94, Benazir-12
ESTR (μM/min/g s. wt.)	≤502	AS-2002, NR-234, UFAQ 2002, 9244, SH-2002, LU-26, Millat-2011, Margalla-99, WL-711, Inqulab-91	503–707	Sitta, Kiran- 95, S-24, Sassui-2006, Bakhtawar-1993, AARI-2011, Saleem-2000, PAVON, Nia Sunhari, Bhakkar-2000	708–988	Punjab-96, Zardana, Raskoh-2005 Mehran-89, Pasban-90, Sarsabz, Fareed-2006, 2156, C-228, Faisalabad-2008
PROT (Units/g s. wt.)	≤6,000	SA-75, Pasban-90, Marvi Mehran-89, Galaxy-2013 Punjab-90, 9021, Bhakkar-2000, Nifa- Bathoor, Zardana	6,001–8,800	2005, Sitta, Watan-94, Takbeer-2000, TC-4928, PAVON, LU-26, Pari-73, SH-2002, Punjab-96	8,801–11,183	BARS-2009, Millat-2011, Nia Sunhari, 6544-6, IQBAL-2000, Jauhar-78, Nia Amber, Chakwal-50, Suleman, NR-234
AA (mg/g s. wt.)	≤200	Dharabi 2011, AARI-2011, UFAQ 2002, Abadghar-93, AUQAB-2000, Jauhar-78, Nia Sunhari, Sassui-2006, Soghat-90, Sehar-2006	201–280	Sarsabz, Margalla-99, Tatara 1996, SA-75, BARS-2009, LU-26, AS-2002, MEXI PAK Punjab-90, C-228	281–293	Takbeer-2000, Khirman2006, Punjab-96, Sindh-81, Galaxy-2013, Pasban-90, Ujala-16, Benazir-12, Lasani-2008, NR-421
TOS (μM/g s. wt.)	≤250	NR-421, Punjab-2011, Nia Sundar, Punjab-90 Takbeer-2000,TC-4928 Ujala-16, SA-75, 2005 Pasban-90	251–291	2006, Kohistan, Punjab-96, Pari-73, C-591, Manthar-2003 Soghat-90, Nifa- Bathoor, Sehar-2006, 9244	290–390	Galaxy-2013, Tatara 1996, Zardana, Sarsabz, Barani-83, NR-234, C-228, Benazir-12, Mehran-89, Nifa Lalma 2012
MDA (μM/g s. wt.)	≤500	PERWAZ, PAVON, Khirman2006, Bakhtawar-1993, Sehar-2006, Pari-73 2005, 6544-6, UFAQ 2002, AS-2002	501–600	MEXI PAK, AUQAB-2000, Punjab-96, Shafaq-2006 Ujala-16, TC-4928, Raskoh-2005, Punjab-2011, Mairaj-2008, AARI-2011	601–679	SA-75, 9021, Takbeer-2000, Benazir-12, NARC-2011, Marvi, Nifa- Bathoor, Mehran-89, C-228, 2006
RS (mg/g s. wt.)	≤4.9	MEXI PAK, Sehar-2006, Dharabi 2011, Mehran-89, Ujala-16, Nifa Lalma 2012, UFAQ 2002, Saleem-2000, 2005, Mairaj-2008	5–8.9	Abadghar-93, Jauhar-78, NR-234, NR-421, AS-2002, Pari-73, Raskoh-2005, Watan-94, Fakhar-e- Sarhad, Marvi	9–13	Nia Sundar, Fareed-2006, LU-26, SA-75, PAVON, IQBAL-2000, Nia Sunhari, Manthar-2003, TC-4928, Sitta
TSS (mg/g s. wt.)	≤11.9	Soghat-90, NR-234, Nifa Lalma 2012, AUQAB-2000, GA-2002, Shafaq-2006, AS-2002, BARS-2009, Mairaj-2008, Inqulab-91	12–15.9	Tatara 1996, Punjab-2011, Fakhar-e- Sarhad, Kohistan, Ujala-16, Fareed-2006, C-228, IQBAL-2000, Jauhar-78 Manthar-2003	16–30	Nesser, Benazir-12, Lasani-2008, LU-26, Bakhtawar-1993, Barani-83 Chakwal-50, Punjab-96, Suleman, Millat-2011
NRS (mg/g s. wt)	≤5.9	Manthar-2003, IQBAL-2000, Sitta, Fareed-2006, Shafaq-2006, TC-4928, NR-234, PERWAZ, Lasani-2008, 6544-6	6–10.9	Inqulab-91, Nia Sundar, Fakhar-e- Sarhad, Sassui-2006, Nifa- Bathoor, BARS-2009, Nifa Lalma 2012, Tatara 1996, Jauhar-78, SH-2002	11–28	Faisalabad-2008, 2005, 2006, PAVON, Barani-83, Benazir-12, NR-421, Millat-2011, Pari-73, Suleman
TSP (mg/g s. wt.)	≤298.0	MEXI PAK, Nesser, Sehar-2006, 2005, Manthar-2003, Benazir-12 Kohistan, Dharabi 2011, PAVON, Sitta	299–400	Fareed-2006, AARI-2011, IQBAL-2000, Soghat-90, PERWAZ, Chakwal-50, Abadghar-93, 2006, NARC-2011, Bhittai-2004	401–487	Pasban-90, SA-75, WL-711, Nifa- Bathoor, Punjab-90, S-24, Pari-73, Punjab-96
ALB (mg/g s. wt.)	≤250	Manthar-2003, Shafaq-2006, Galaxy-2013, Bakhtawar-1993, Nia Amber, LU-26, SA-75, Punjab-2011, Tatara 1996, Fareed-2006	251–290	Lasani-2008, MEXI PAK, Sehar-2006, Pasban-90, Suleman, Nesser, PERWAZ, Chakwal-50, Marvi, 2006	291–353	Millat-2011, Dharabi 2011, Nia Sunhari, 2156, AS-2002, WL-711, Benazir-12, Takbeer-2000, PAVON, Punjab-96
GLOB (mg/g s. wt.)	≤161	Nifa Lalma 2012, BARS-2009, Saleem-2000, Sindh-81, GA-2002, Sarsabz, PAVON, SH-2002, Khirman2006, Inqulab-91	162–199.9	Nia Amber, Raskoh-2005, Sassui-2006, Punjab-2011, Benazir-12, Nia Sundar, Margalla-99, PERWAZ, Mehran-89, NR-234	200–253	AARI-2011, Soghat-90, Pari-73 LU-26, WL-711, Watan-94, Fakhar-e- Sarhad, Lasani-2008, Millat-2011, Tatara 1996
SSP (mg/g s. wt.)	≤70	Punjab-90, 6544-6, Shafaq-2006, Saleem-2000, NR-234, Sitta, Nia Sunhari, Barani-83, 9021, S-24	71–110	2006, IQBAL-2000, 2156, Inqulab-91, Raskoh-2005, Kohistan, Nia Sundar, Punjab-2011, Sindh-81, Pari-73	111–162	9244, Nifa Lalma 2012, SA-75, C-591, Soghat-90, Jauhar-78, Sehar-2006, Galaxy-2013, MEXI PAK, Millat-2011

*SOD, Superoxide Dismutase; CAT, Catalase; APX, Ascorbate Peroxidase; POD, Peroxidase; TPC, Total Phenolic Content; AsA, Ascorbic Acid; ESTR, Esterase; PROT, Protease; AA, Alpha Amylase; TOS, Total Oxidant Status; MDA, Malondialdehyde; RS, Reducing Sugars; TSS, Total Soluble Sugars; NRS, Non-Reducing Sugars; TSP, Total Soluble Protein; ALB, Albumin; GLOB, Globulin; SSP, Salt Soluble Protein; Seed Weight, S. Wt*.

#### Ascorbate peroxidase (APX) activity

Genotypes were divided in three categories based on comparative values of different studied parameters (Table 2). For APX activity in wheat seeds, significant variation was found among wheat genotypes and it was possible to categorize them in low, medium, and high classes. Five genotypes were grouped in high category for APX, in which the values ranged from 1,000 to 1,427 Units/g s. wt. Mean values for APX are presented as Figure S3. Among these genotypes highest APX activity was found in Pavon (1,426.67 Units/g s. wt.). Forty-nine genotypes with APX activity ranging from 401 to 999 Units/g s. wt. were grouped as intermediate class. Twenty-three genotypes were grouped in third category, that is genotypes having comparatively low APX activity. In this class, genotypes having APX activity <400 Units/g s. wt. were grouped. Among these genotypes having comparatively low APX activity, the lowest value was observed in NARC-2011 (173.33 Units/g s. wt.).

#### Peroxidase (POD) activity

Genotypes were divided in three categories based on comparative values of different studied parameters (Table 2). For peroxidase activity in wheat seeds, significant variation was found among wheat genotypes and it was possible to categorize them in low, medium, and high classes. Seven genotypes were grouped in high category for POD, in which the values ranged from 20,000 to 42,580 Units/g s. wt. Mean values for POD are presented as Figure S4. Among these genotypes highest POD activity was found in IQBAL-2000 (42,579.6 Units/g s. wt.). Twenty-four genotypes with POD activity ranging from 9,001 to 19,999 Units/g s. wt. were grouped as intermediate class. Forty-six genotypes were grouped in third category that is genotypes having comparatively low POD activity. In this class genotypes having POD activity <9,000 Units/g s. wt. were grouped. Among these genotypes having comparatively low POD activity, the lowest value was observed in Punjab-90 (1,642.8 Units/g s. wt.).

### Non-enzymatic antioxidants

#### Total phenolic content (TPC)

Genotypes were divided in three categories based on comparative values of different studied parameters (Table 2). For total phenolic content in wheat seeds, significant variation was found among wheat genotypes and it was possible to categorize them in low, medium, and high classes. Fourteen genotypes were grouped in high category for TPC, in which the values ranged from 20,233 to 25,383 (μM/g s. wt.). Mean values for TPC are presented as Figure S5. Among these genotypes highest TPC was found in Bhakkar-2000 (25,383.33 μM/g s. wt.). Fifty genotypes with TPC ranging from 9,701 to 20,232 (μM/g s. wt.) were grouped as intermediate class. Thirteen genotypes were grouped in third category that is genotypes having comparatively low TPC. In this class genotypes having TPC <9,700 (μM/g s. wt.) were grouped. Among these genotypes having comparatively low TPC, the lowest value was observed in WL-711 (1,200.0 μM/g s. wt.).

#### Ascorbic acid (AsA) content

Genotypes were divided in three categories based on comparative values of different studied parameters (Table 2). For ascorbic acid content in wheat seeds, significant variation was found among wheat genotypes and it was possible to categorize them in low, medium, and high classes. Thirteen genotypes were grouped in high category for ascorbic acid content, in which the values ranged from 682 to 713 μg/g s. wt. Mean values for AsA are presented as Figure S6. Among these genotypes, highest ascorbic acid content was found in SH-2002 (713.0 μg/g s. wt.). Fifty genotypes with ascorbic acid content ranging from 641 to 681 μg/g s. wt. were grouped as intermediate class. Thirteen genotypes were grouped in third category that is genotypes having comparatively low ascorbic acid content. In this class genotypes having ascorbic acid content <640 μg/g s. wt. were grouped. Among these genotypes having comparatively low ascorbic acid content, the lowest value was observed in S-24 (439.5 μg/g s. wt.).

### Hydrolytic enzymes

#### Esterase activity

Genotypes were divided in three categories based on comparative values of different studied parameters (Table 2). Seventeen genotypes were grouped in high category for esterase activity, in which the values ranged from 708 to 988 μM/min/g s. wt. Mean values for esterase activity are presented as Figure S7. Among these genotypes highest esterase activity was found in Dharabi 2011 (987.80 μM/min/g s. wt.). Thirty genotypes with esterase activity ranging from 503 to 707 μM/min/g s. wt. were grouped as intermediate class. Thirty genotypes were grouped in third category that is genotypes having comparatively low esterase activity. In this class, genotypes having esterase activity <502 μM/min/g s. wt. were grouped. Among these genotypes having comparatively low esterase activity, the lowest value was observed in AS-2002 (358.53 μM/min/g s. wt.).

#### Protease activity

Genotypes were divided in three categories based on comparative values of different studied parameters (Table 2). For protease activity, in wheat seeds, significant variation was found among wheat genotypes and it was possible to categorize them in low, medium, and high classes. Ten genotypes were grouped in high category for protease activity, in which the values ranged from 8,801 to 11,183 Units/g s. wt. Mean values for protease activity are presented as Figure S8. Among these genotypes, highest protease activity was found in NR-234 (11,183.33 Units/g s. wt.). Fifty-three genotypes with protease activity ranging from 6,001 to 8,800 Units/g s. wt. were grouped as intermediate class. Fourteen genotypes were grouped in third category that is genotypes having comparatively low protease activity. In this class genotypes having protease activity <6,000 Units/g s. wt. were grouped. Among these genotypes having comparatively low protease activity, the lowest value was observed in SA-75 (4,023.33 Units/g s. wt.).

#### Alpha amylase activity

Genotypes were divided in three categories based on comparative values of different studied parameters (Table 2).

For alpha amylase activity in wheat seeds, significant variation was found among wheat genotypes and it was possible to categorize them in low, medium, and high classes. Twelve genotypes were grouped in high category for α-amylase, in which the values ranged from 281 to 293 (mg/g s. wt.). Mean values for α-amylase activity are presented as Figure S9. Among these genotypes highest α-amylase activity was found in SH-2002 (292.70 mg/g s. wt.). Twenty-eight genotypes with α-amylase activity ranging from 201 to 280 mg/g s. wt.) were grouped as intermediate class. Thirty-seven genotypes were grouped in third category that is genotypes having comparatively low α-amylase activity. In this class genotypes having α-amylase activity <200 mg/g s. wt. were grouped. Among these genotypes having comparatively low α-amylase activity, the lowest value was observed in Dharabi 2011 (112.58 mg/g s. wt.).

### Other biochemical parameters

#### Total oxidant status (TOS)

Genotypes were divided in three categories based on comparative values of different studied parameters (Table 2). For TOS in wheat seeds, significant variation was found among wheat genotypes and it was possible to categorize them in low, medium, and high classes. Seventeen genotypes were grouped in high category for TOS, in which the values ranged from 290 to 390 μM/g s. wt. Mean values for TOS are presented as Figure S10. Among these genotypes highest TOS was found in Faisalabad-2008 (390.0 μM/g s. wt.). Thirty genotypes with TOS ranging from 251 to 291 μM/g s. wt. were grouped as intermediate class. Thirty genotypes were grouped in third category that is genotypes having comparatively low TOS. In this class genotypes having TOS <250 μM/g s. wt. were grouped. Among these genotypes having comparatively low TOS, the lowest value was observed in NR-421 (241.0 μM/g s. wt.).

#### Malondialdehyde (MDA) content

Genotypes were divided in three categories based on comparative values of different studied parameters (Table 2). For MDA content (μM/g s. wt.) in wheat seeds, significant variation was found among wheat genotypes and it was possible to categorize them in low, medium, and high classes. Thirteen genotypes were grouped in high category for MDA, in which the values ranged from 601 to 679 μM/g s. wt. Mean values for MDA are presented as Figure S11. Among these genotypes highest MDA activity was found in Margalla-99 (679.23 μM/g s. wt.). Twenty-eight genotypes with MDA activity ranging from 501 to 600 μM/g s. wt. were grouped as intermediate class. Thirty-six genotypes were grouped in third category that is genotypes having comparatively low POD activity. In this class genotypes having MDA activity <500 μM/g s. wt. were grouped. Among these genotypes having comparatively low MDA activity, the lowest value was observed in PERWAZ (167.23 μM/g s. wt.).

#### Reducing sugars

Genotypes were divided in three categories based on comparative values of different studied parameters (Table 2). Twelve genotypes were grouped in high category for reducing sugars, in which the values ranged from 9 to 13 mg/g s. wt. Mean values for reducing sugars are presented as Figure S12. Among these genotypes highest reducing sugars was found in Punjab-96 (12.68 mg/g s. wt.). Twenty-four genotypes with reducing sugars ranging from 5 to 8.9 mg/g s. wt. were grouped as intermediate class. Forty-one genotypes were grouped in third category that is genotypes having comparatively low reducing sugars. In this class genotypes having reducing sugars <4.9 mg/g s. wt. were grouped. Among these genotypes having comparatively low reducing sugars, the lowest value was observed in MEXI PAK (1.27 mg/g s. wt.).

#### Total soluble sugars

Genotypes were divided in three categories based on comparative values of different studied parameters (Table 2). For total soluble sugars in wheat seeds, significant variation was found among wheat genotypes and it was possible to categorize them in low, medium, and high classes. Twenty-four genotypes were grouped in high category for total soluble sugars, in which the values ranged from 16 to 30 mg/g s. wt.) Mean values for total soluble sugars are presented as Figure S13. Among these genotypes highest total soluble sugars was found in Saleem-2000 (29.86 mg/g s. wt.). Thirty-three genotypes with total soluble sugars ranging from 12 to 15.9 mg/g s. wt. were grouped as intermediate class. Twenty genotypes were grouped in third category that is genotypes having comparatively low total soluble sugars. In this class genotypes having total soluble sugars <11.9 mg/g s. wt. were grouped. Among these genotypes having comparatively low total soluble sugars, the lowest value was observed in Soghat-90 (7.77 mg/g s. wt.).

#### Non-reducing sugars

Genotypes were divided in three categories based on comparative values of different studied parameters (Table 2). For non-reducing sugars in wheat seeds, significant variation was found among wheat genotypes and it was possible to categorize them in low, medium, and high classes. Twenty-three genotypes were grouped in high category for non-reducing sugars, in which the values ranged from 11 to 28 mg/g s. wt. Mean values for non-reducing sugars are presented as Figure S14. Among these genotypes highest non-reducing sugars was found in Saleem-2000 (27.33 mg/g s. wt.). Thirty-five genotypes with non-reducing sugars ranging from 6 to 10.9 mg/g s. wt. were grouped as intermediate class. Nineteen genotypes were grouped in third category that is genotypes having comparatively low non-reducing sugars. In this class, genotypes having non-reducing sugars <5.9 mg/g s. wt. were grouped. Among these genotypes having comparatively low non-reducing sugars, the lowest value was observed in Manthar-2003 (1.31 mg/g s. wt.).

### Differential protein estimation

#### Total soluble protein

Genotypes were divided in three categories based on comparative values of different studied parameters (Table 1). Eight genotypes were grouped in high category for total soluble proteins, in which the values ranged from 401 to 487 mg/g s. wt. Mean values for total soluble proteins are presented as Figure S15. Among these genotypes highest total soluble proteins was found in Punjab-96 (487.33 mg/g s. wt.). Twenty-nine genotypes with total soluble proteins ranging from 299 to 400 mg/g s. wt. were grouped as intermediate class. Forty genotypes were grouped in third category that is genotypes having comparatively low total soluble proteins. In this class, genotypes having total soluble proteins <298.0 mg/g s. wt. were grouped. Among these genotypes having comparatively low total soluble proteins, the lowest value was observed in MEXI PAK (68.67 mg/g s. wt.).

#### Albumins

Genotypes were divided in three categories based on comparative values of different studied parameters (Table 2). Twenty-one genotypes were grouped in high category for albumins, in which the values ranged from 291 to 353 mg/g s. wt. Mean values for albumins are presented as Figure S16. Among these genotypes highest albumins was found in TC-4928 (352.89 mg/g s. wt.). Thirty-three genotypes with albumins ranging from 251 to 290 mg/g s. wt. were grouped as intermediate class. Twenty-three genotypes were grouped in third category that is genotypes having comparatively low albumins. In this class, genotypes having albumins <250 mg/g s. wt. were grouped. Among these genotypes having comparatively low albumins, the lowest value was observed in Manthar-2003 (199.56 mg/g s. wt.).

#### Globulins

Genotypes were divided in three categories based on comparative values of different studied parameters (Table 2). Eleven genotypes were grouped in high category for globulins, in which the values ranged from 200 to 253 mg/g s. wt. Mean values for globulins are presented as Figure S17. Among these genotypes highest globulins was found in MEXI PAK (252.67 mg/g s. wt.). Thirty-two genotypes with globulins ranging from 162 to 199.9 mg/g s. wt. were grouped as intermediate class. Thirty-four genotypes were grouped in third category that is genotypes having comparatively low globulins. In this class, genotypes having globulins <161 mg/g s. wt. were grouped. Among these genotypes having comparatively low globulins, the lowest value was observed in Nifa Lalma 2012 (112.44 mg/g s. wt.).

#### Salt soluble protein

Genotypes were divided in three categories based on comparative values of different studied parameters (Table 2). Seventeen genotypes were grouped in high category for salt soluble proteins, in which the values ranged from 111 to 162 mg/g s. wt. Mean values for salt soluble proteins are presented as Figure S18. Among these genotypes highest salt soluble proteins was found in Faisalabad-2008 (162.44 mg/g s. wt.). Forty genotypes with salt soluble proteins ranging from 71 to 110 mg/g s. wt. were grouped as intermediate class. Twenty genotypes were grouped in third category that is genotypes having comparatively low salt soluble proteins. In this class, genotypes having salt soluble proteins <70 mg/g s. wt. were grouped. Among these genotypes having comparatively low salt soluble proteins, the lowest value was observed in Punjab-90 (61.33 mg/g s. wt.).

### Principal component analysis

**Figure 1:**
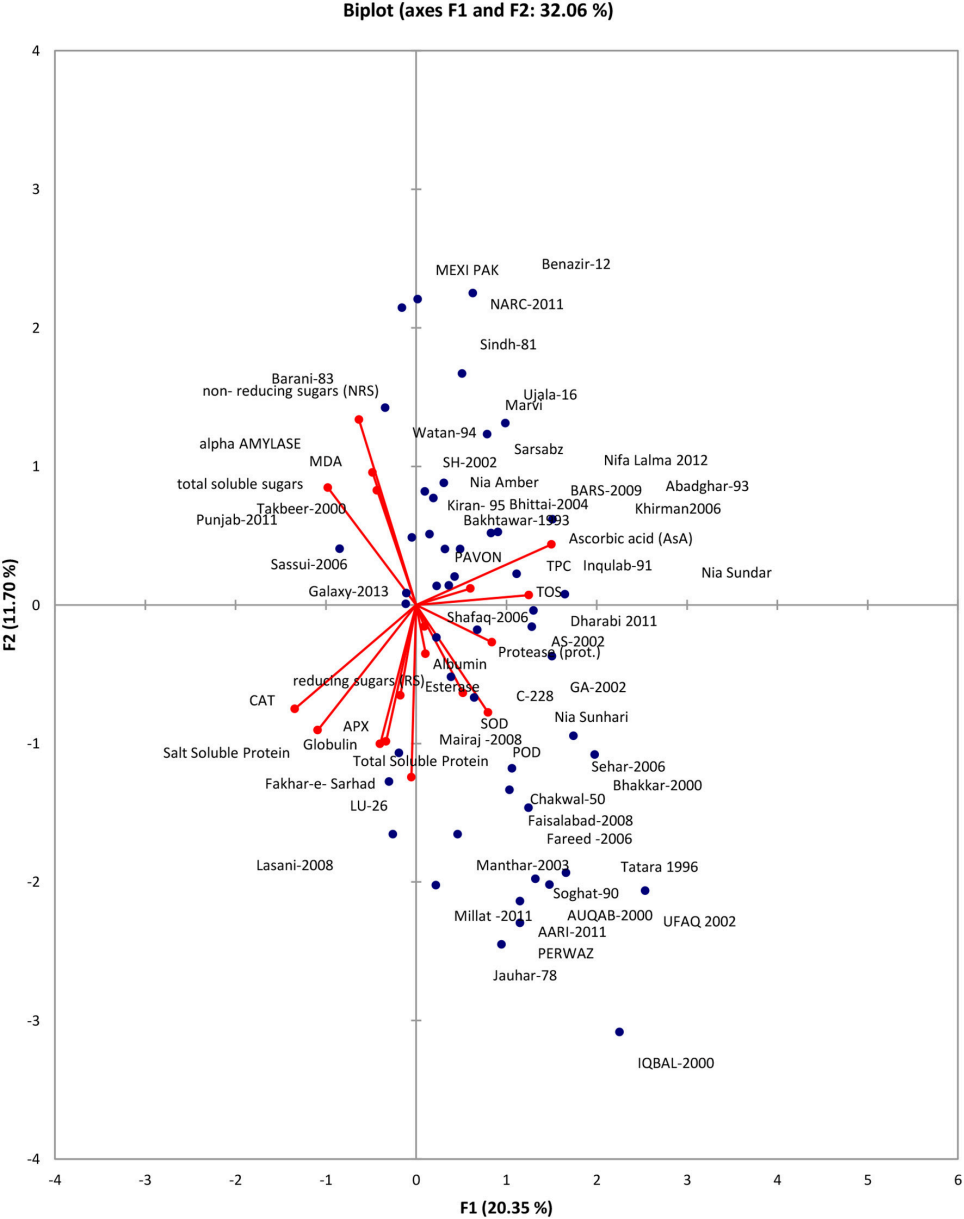
Bi-plot of wheat genotypes for first two principal components.

Data was subjected to principal component analysis. Out of the 18 principal components PC(s), seven viz. PC-1, PC-II, PC-III, PC-IV, PC-V, PC-VI, PC-VII had Eigenvalues >1 and contributed for 69.88% of total cumulative variability among different genotypes (Table S1). The contribution of PC-I toward variability was highest (20.35%) followed by PC-II, PC-III, PC-IV, PC-V, PC-VI, PC-VII which contributed 11.71% and 10.46, 8.37, 6.81, 6.52, and 5.68% variability, respectively. The biplot depicted overall association of wheat genotypes for 18 traits (Figure 1). The first two principal components who contributed 32.06% toward total variance were plotted on PC-I x axis and PC-II on y-axis to detect the association between different clusters. The genotype by trait (G-T) biplot thus described 32.06% of the total variation. In G-T biplot, a vector was drawn from origin to every trait which enables the visualization of inter-relationships among characters.

## Discussion

Wheat is the most extensively cultivated cereal grain around the globe and holds crucial place in agriculture (Kumar et al., 2013; Nawaz et al., 2013). It is a principal nutriment for 35% of the world's populace. In Pakistan, it covers 70% of Rabbi and 37% of entire cropped sector (Khan et al., 2011). A number of studies have reported the effect of genotype, growing conditions, and interactions between the environment and the genotype on the antioxidant properties of wheat. While in the efforts for increasing yield and disease resistance, the quality attributes might have been compromised. In this study, efforts were made to explore the biochemical composition of different wheat varieties for food and nutritional purposes, as the breeding efforts remained focused on increasing yield and quality enhancements remained unexploited. For this purpose, wheat varieties were selected from several/diverse centers in each agro-climatic zone of Pakistan for testing the grain biochemical composition and antioxidant potential. Detailed investigation on wheat seed biochemical profiling of Pakistani genotypes was missing so far. To our knowledge, for the first time Pakistani wheat genotypes have been categorized based on seed biochemical profiles.

In present study, total varieties released during 1990's were 31 while during 2000 and later on were 36 whereas rest of the 10 were advance lines. Ascorbate peroxidase (APX) enzyme is an essential component of the glutathione ascorbate cycle (Noctor and Foyer, 1998) and is involved in the detoxification of hydrogen peroxide. It is involved in the protection of plant cells from environmental stress (Zhang, 2013). It was found that 31 and 69.4% of the genotypes released during 2000 to onward have low and intermediate APX activity while 16.1% of the varieties released during 1990's showed highest APX activity. In general, APX activity was found highest in PAVON (1,426.67 Units/g s. wt.).

Catalase catalyses the hydrogen peroxide conversion to water and oxygen with the help of cofactor iron or manganese (Chelikani et al., 2004). Catalase is involved in oxidative reaction during bread making, bleaching of pigment and prevents the accretion of harmful hydrogen peroxide (Kruger and Leberg, 1974). The expression of specific catalase isoenzymes is important and critical against oxidative stress induced by a given environmental stress (Bakalova et al., 2004). Seed filling is allied with the high potential of the hydrogen peroxide detoxification machinery, primarily due to CAT and APX activities (Ishibashi et al., 2008). In 42% of the genotypes released during the year 2000 and onward, least CAT activity was found while intermediate activity was detected in 61% of the genotypes which were released during the year 2000 and onward whereas 10% of the genotypes released during 1990's hold higher CAT activity. Overall, CAT activity was found highest in Pasban-90 (633.33 Units/g s. wt.).

Peroxidase (POD) aids in scavenging the reactive oxygen species which are responsible for cell oxidative injury (Vicuna, 2005). Plant peroxidases have been used as biochemical markers for numerous types of abiotic as well as abiotic stresses because of their role in very imperative physiological processes, like control of growth by lignification, cross-linking of pectins and structural proteins in cell wall, catabolism of auxins (Bakalova et al., 2004). Lowest POD activity was found in 71% of genotypes released during the year 1990 and onward while 42% of the genotypes released during 2000 and onward showed intermediate POD activity, whereas POD activity was found highest in 14% of the genotypes released during 2000 and onwards. In general, highest POD activity was found in IQBAL-2000 (42,579.6 Units/g s. wt.).

Malondialdehdye is the ultimate result of oxidative degradation which causes alteration in membrane characteristic such as loss of enzyme activity (Sharma et al., 2012). Malondialdehdye signifies the level of damage to the plant cells (Zhang, 2013). Lipid peroxidation is main cause of seed injury in the course of storage and is responsible for initial biochemical alterations in seed which can be detected during storage. Increased content of MDA is associated with prolonged storage period and accelerating aging (Goodarzian et al., 2014). Least MDA content was found in 56% of the genotypes released during 2000 and onwards and intermediate content was shown by the 42% of genotypes released during 1990's whereas it was found highest in 16.1% of the genotypes released during 1990's and onward. In general, highest MDA activity was found in Margalla-99 (679.23 μM/g s. wt.).

Alpha amylase is involved in absorption properties, gassing power of dough and in final properties of bread (Freeman and Ford, 1941). Alpha amylase activity was low in 47.2% of genotypes released during 2000 and onwards while it was found intermediate in 65% of the genotypes released during 1990's. However, 22.2% of the genotypes showed highest alpha amylase activity. In general, highest alpha amylase activity was found in SH-2002 (292.70 mg/g s. wt.).

Superoxide dismutase (SOD) enzymes act as antioxidant and protect the cellular components oxidation through reactive oxygen species (Alscher et al., 2002). In 32.2% of the genotypes released during 1990's, lowest SOD activity was found whereas 19.4% of the genotypes released during 2000 and onwards showed intermediate activity while it was found highest in 55.6% of the genotypes released during 2000 and onward. Overall, highest SOD activity was found in Manthar-2003 (278.93 Units/g s. wt.). Esterase activity was lower in 42% of the genotypes released during 1990's whereas 56% of genotypes released during 2000 and onwards revealed intermediate activity. Highest esterase activity was found in 29.03% of the genotypes released during 1990's. As a whole, highest esterase activity was found in Dharabi 2011 (987.80 μM/min/g s. wt.).

Total oxidant status (TOS) was lower or intermediate in 11.1 and 28% of the genotypes, respectively, released during 2000 and onward while it was found highest in 58.1% of the genotypes released during 1990's. In general, highest TOS was found in Faisalabad-2008 (390.0 μM/g s. wt.).

Wheat grains are rich in phenolic compounds with potential health benefits. Phenolic compounds also contribute directly to anti oxidative action (Awika et al., 2003). Total phenolic content (TPC) was low in 23% of the genotypes released during 1990s, while 67% of the genotypes showed intermediate content, released during 2000 and highest phenolic content was found in 25% of the genotypes released during 2000. On the whole, highest TPC was found in Bhakkar-2000 (25,383.33 μM/g s. wt.). In a previous finding, total phenolic content (709.8–860.0 μM of gallic acid equiv/100 g of wheat) did not vary greatly in 11 tested wheat varieties (Adom et al., 2003). In another study, significant effect of the bread-making process with the TPC of whole wheat bread (1.50–1.65 mg/g) was found while comparing five brands of whole and refined wheat flour (Yu and Nanguet, 2013). Total phenolic acid content varied from 2,900 to 5,650 μg GAE/g in six Indian wheat varieties (Narwal et al., 2014). All the bran extracts showed considerable total phenolic content (2.12–3.37 mg Gallic acid equivalent/g bran) while evaluating bran's extract antioxidant potential from five genotypes of wheat native to Pakistan (Iqbal et al., 2007). Although number of studies have described the phenolic content in wheat varieties, however, comparison of results is not possible in most of cases because diverse procedures for extraction as well as different solvents and evaluating methods have been used.

Ascorbic acid interacts directly with superoxide anion radical and hydroxyl radical. It helps to minimize the consequences of lipid peroxidation (Gupta and Sharma, 2006). Ascorbic acid acts as a plant growth modulator through hormone signaling. It has significant role in many physiological processes like seed germination, flowering, permeability of membrane, growth of seedlings, intake of ions into roots, respiration, photosynthesis, senescence, protein and content of nucleic acid, and enzymes activities for example peroxidase and SOD (Cavusoglu and Bilir, 2015). Ascorbic acid content was found low in 36% of the genotypes released during 1990's. Intermediate content of ascorbic acid was found in 33.3% of the genotypes released during the year 2000 and onwards and it was found highest in 58.3% genotypes. Overall, highest ascorbic acid content was found in SH-2002 (713.0 μg/g s. wt.).

Proteolytic enzymes play an essential role in physiology as well as development of plants and work as a source of amino acids required for the production of novel proteins. (Schaller, 2004). Protease activity was lower in 19.4% of the genotypes released during the 1990's while intermediate in 69.4% of the genotypes released during 2000 and onward it was found highest in 17% of the genotypes released during 2000 and onwards. In general, highest protease activity was found in NR-234 (11,183.33 Units/g s. wt.).

Total soluble sugars were lower in 31% of the genotypes released during 2000 and above while they were found intermediate in 42% of the genotypes released during 2000 and onwards. Highest total soluble sugars were detected in 45.2% of the genotypes released during 1990's. In general, highest total soluble sugars were found in Saleem-2000 (29.86 mg/g s. wt.). Reducing sugars were found low in 58.3% of the genotypes released during 2000 and on wards whereas these were found intermediate in 36% of the genotypes released during 1990's and reducing sugars were higher in 17% of the genotypes released during 2000. On the whole, highest reducing sugars were found in Punjab-96 (12.68 mg/g s. wt.).

Endosperm of wheat comprises about 20–25% albumins and globulins of total proteins. They are soluble in water, biologically active, and have role in metabolism (Merlino et al., 2009; Khan et al., 2015). Although they are functional proteins but have nutritional importance as well but also possess allergenic effects (Khan et al., 2013). In a previous report, genotype Sindh-90 has been reported to contain the highest content of albumin i.e., 22.4% and albumin besides globulin accounts for 23.78 to 27.26% of total proteins (Khan et al., 2013). The grains quality can be estimated from percentage of protein which exists in them. Albumins and globulins act as nutrient reserves for the sprouting embryo and also help in protecting embryo from insects and pathogens prior to germination (Malik, 2009). In our study, albumins were found low in 36.11% of the genotypes released during 2000 and onwards while 48.4% of the genotypes released during 1990's showed intermediate albumins whereas highest albumins were detected in 31% of the genotypes released during 1990's. Overall, highest albumins were found in TC-4928 (352.89 mg/g s. wt.).

Globulins were found low and intermediate in 50 and 44.4% of the genotypes, respectively, released during 2000 and onwards while detected to be highest in 36% of the genotypes released during 1990's. In general, highest globulins were found in MEXI PAK (252.67 mg/g s. wt.). In a previous study on Pakistani wheat varieties, globulin protein was reported be ~3–7.0 and 9.15–13.80% (Ikhtiar and Alam, 2007). whereas our results showed higher protein content with albumins and globulins representing about 25–35% of the total protein and these findings are consistent with the previous one (Khan et al., 2015). Many factors like fertilizers, environmental and storage condition, soil and location, etc. can influence the seed protein content and cause variation in it (Khan et al., 2013).

Salt soluble protein were lower in 26% of the genotypes released during 1990's while these were found intermediate in 61.1% of the genotypes released during 2000 and on wards and 26% of the genotypes showed highest content of salt soluble protein. Overall, highest salt soluble proteins was found in Faisalabad-2008 (162.44 mg/g f. wt.).

Wheat seed storage proteins play a vital role in determining quality as dough extensibility and elasticity is because of them (Rasheed et al., 2014). Total soluble proteins were found low to intermediate in 50% of the genotypes released during 2000 and onward while 19.4% of the genotypes released during 1990's showed higher content of total soluble proteins. As a whole, highest total soluble proteins were found in Punjab-96 (487.33 mg/g s. wt.). Present results are within range as reported in previously where storage proteins are almost 50 and 8–20% of the entire protein in mature grains of cereals (wheat) (Shewry and Halford, 2002; Khan et al., 2013). Wheat proteins are also responsible for food related allergens as well as intolerances (Rasheed et al., 2014) therefore it can be inferred from results that people suffering from wheat related sensitivity should avoid using wheat based food products rich in proteins.

Non-reducing sugars were lower in 28% of the genotypes released during 2000 and onward whereas these were intermediate in 18% of the genotypes released during 2000 and onward and 36% of the genotypes showed highest content of non-reducing sugars. In general, highest non-reducing sugar was found in Saleem-2000 (27.33 mg/g s. wt.).

Highest TPC and POD activity was found in genotypes IQBAL-2000 (drought tolerant) (Noorka et al., 2013) and BHAKKAR-2000, respectively, (salt tolerant) (Zafar et al., 2015) and both varieties were released during 2000 by AZRI, Bhakkar. While highest amount of salt soluble protein was found in genotype Faisalabad-2008 (drought tolerant) (Noorka et al., 2013) released during 2008 by AARI, Faisalabad whereas reducing sugars and total soluble protein was found highest in genotype Punjab-96 (drought tolerant) (Ahmad et al., 2003) released during 1996 by AARI, Faisalabad. Genotype Saleem-2000 showed maximum TSS and NRS, released during 2001 by CCRI, Pirsabak whereas APX activity was found highest in PAVON released during 1978 by AARI, Faisalabad. However, Pasban-90 (drought tolerant) (Ahmad et al., 2003) showed highest CAT activity, released during the year 1991 by AARI, Faisalabad while, highest activity of SOD was found in Manthar-2003 (drought tolerant) (Bano and Yasmeen, 2010) released during 2003 by RARI, Bahawalpur. Maximum esterase activity was found in Dharabi-2011 (drought tolerant) (Tariq et al., 2013) released during 2011 by BARI, Chakwal and protease activity was maximum in advance line NR-234 from, NARC, Islamabad but MDA content was highest in Margalla-99 (drought and salt tolerant) (Bano and Yasmeen, 2010; Zafar et al., 2015) released during 1999 by NARC, Islamabad. Highest alpha amylase activity and ascorbic acid was found in SH-2002 (drought tolerant) (Noorka et al., 2013) released during 2002 by AARI, Faisalabad. Albumin was highest in TC-4928 advance line from UAF whereas globulin was maximum in MEXI PAK released during 1965 by AARI, Faisalabad. All these observations pointed out that highest values of studied parameters especially those related to stress response were observed in genotypes reported to be tolerant for stresses. It can be thus deduced that higher levels of these stress responsive attributes in seeds of these genotypes play some important role in stress tolerant phenotypes even at later growth stages.

Principal component analysis reveals the chief contributor's significance to the overall variation at each differentiation axis. The Eigenvalues assist in defining the total factors which can be retained. Sum of the Eigenvalues is generally equivalent to the amount of variables (Bhanupriya et al., 2014). Numerals with highest absolute value nearer to unity in the first principal component effects the grouping more in comparison to those with lesser absolute value nearer to zero (Bhanupriya et al., 2014; Mishra et al., 2015). In present study, out of the 18 principal components PC(s), seven viz. PC-1, PC-II, PC-III, PC-IV, PC-V, PC-VI, PC-VII had Eigenvalues >1 and contributed for 69.88% of total cumulative variability among different genotypes. The contribution of PC-I toward variability was highest (20.35%). The PC-I showed positive factor loadings for POD, SOD, esterase, TOS, TPC, Protease, AsA, and albumin while PC-II indicated positive factor loading for MDA, alpha amylase, TOS, TPC, AsA, NRS, and TSS. Usually, one variable is chosen from these recognized clusters depending on individual loadings (Mishra et al., 2015). Hence, AsA, a non-enzymatic antioxidant is the finest choice which has highest contribution to PC-I whereas NRS a carbohydrate, was the chief contributor to PC-II and NRS in PC-III. These results clearly showed that PC (s) analysis, in parallel to genetic resource characterization also pointed out particular traits of interest for designing breeding strategies.

## Conclusion

Present findings revealed that selected varieties of wheat from different geographical origins of Pakistan i.e., Pavon (APX), Pasban-90 (CAT), IQBAL-2000 (POD), Manthar-2003 (SOD), SH-2002 (alpha amylase and ascorbic acid), Dharabi 2011 (esterase), NR-234 (protease), Faisalabad-2008 (TOS and salt soluble protein), Margalla-99 (MDA content), Bhakkar-2000 (TPC), Saleem 2000 (total soluble sugars and non-reducing sugars), Punjab-96 (reducing sugars and total soluble protein), TC-4928 (albumins), and MEXI PAK (globulins) have great diversity in terms of nutritional potential (antioxidant potential and protein content). Hence, varieties with maximum nutritional potential can be explored for baking products and for breeding of new cultivars with superior nutritional quality.

## Author contributions

AK contributed in overall execution of the experiment, analytical work, collection of data after biochemical analysis of seeds, organization of resulting data, write up and revision of manuscript. AH contributed in planning, designing and finalization of basic idea of experiment, overall supervision during analytical work, statistical analysis of data, revision and finalization of manuscript.

### Conflict of interest statement

The authors declare that the research was conducted in the absence of any commercial or financial relationships that could be construed as a potential conflict of interest.
