# Supplementary material for: Seed Biochemical Analysis Based Profiling of Diverse Wheat Genetic Resource from Pakistan
Source: Front Plant Sci. 2017 Jul 20;8:1276. doi: 10.3389/fpls.2017.01276 (PMC5517496; doi:10.3389/fpls.2017.01276)
Supplement: Supplementary file 1 [file DataSheet1.DOCX]

**Figure S1:** Mean values for superoxide dismutase activity in seeds of different wheat genotypes.

**Figure S2:** Mean values for catalase activity in seeds of different wheat genotypes.

**Figure S3:** Mean values for ascorbate peroxidase activity in seeds of different wheat genotypes.

**Figure S4:** Mean values for peroxidase activity in seeds of different wheat genotypes.

**Figure S5:** Mean values for total phenolic content in seeds of different wheat genotypes.

**Figure S6:** Mean values for ascorbic acid content in seeds of different wheat genotypes.

**Figure S7:** Mean values for esterase activity in seeds of different wheat genotypes.

**Figure S8:** Mean values for protease activity in seeds of different wheat genotypes.

**Figure S9:** Mean values for alpha amylase activity in seeds of different wheat genotypes.

**Figure S10:** Mean values for total oxidant status in seeds of different wheat genotypes.

**Figure S11:** Mean values for malondialdehyde content in seeds of different wheat genotypes.

**Figure S12:** Mean values for reducing sugars in seeds of different wheat genotypes.

**Figure S13:** Mean values for total soluble sugars in seeds of different wheat genotypes.

**Figure S14:** Mean values for non reducing sugars in seeds of different wheat genotypes.

**Figure S15:** Mean values for total soluble protein in seeds of different wheat genotypes.

**Figure S16:** Mean values for albumins in seeds of different wheat genotypes.

**Figure S17:** Mean values for globulins in seeds of different wheat genotypes.

**Figure S18:** Mean values for salt soluble protein in seeds of different wheat genotypes.

**Table S1. Principal component analysis for different biochemical parameters in wheat genotypes.**

|  | **F1** | **F2** | **F3** | **F4** | **F5** | **F6** | **F7** | **F8** | **F9** | **F10** | **F11** | **F12** | **F13** | **F14** | **F15** | **F16** | **F17** |
| --- | --- | --- | --- | --- | --- | --- | --- | --- | --- | --- | --- | --- | --- | --- | --- | --- | --- |
| **Eigenvalue** | 3.66 | 2.11 | 1.88 | 1.51 | 1.23 | 1.17 | 1.02 | 0.93 | 0.81 | 0.76 | 0.70 | 0.61 | 0.52 | 0.40 | 0.32 | 0.27 | 0.11 |
| **Variability (%)** | 20.35 | 11.71 | 10.46 | 8.37 | 6.81 | 6.52 | 5.68 | 5.19 | 4.48 | 4.21 | 3.89 | 3.39 | 2.90 | 2.19 | 1.78 | 1.48 | 0.62 |
| **Cumulative %** | 20.35 | 32.06 | 42.51 | 50.89 | 57.69 | 64.21 | 69.89 | 75.07 | 79.55 | 83.76 | 87.64 | 91.03 | 93.94 | 96.12 | 97.90 | 99.38 | 100.00 |
| **Eigenvector:**  **variables** | | | | | | | | | | | | | | | | | |
|  | F1 | F2 | F3 | F4 | F5 | F6 | F7 | F8 | F9 | F10 | F11 | F12 | F13 | F14 | F15 | F16 | F17 |
| **SOD** | 0.158 | -0.193 | 0.116 | -0.284 | -0.353 | 0.277 | -0.274 | -0.066 | 0.153 | 0.444 | -0.367 | -0.275 | 0.136 | -0.289 | -0.166 | -0.002 | 0.053 |
| **CAT** | -0.407 | -0.228 | 0.054 | 0.026 | 0.022 | -0.196 | -0.065 | -0.046 | 0.154 | 0.106 | -0.131 | 0.063 | 0.008 | 0.289 | -0.185 | 0.701 | 0.263 |
| **APX** | -0.053 | -0.198 | 0.414 | 0.375 | 0.185 | 0.178 | -0.235 | -0.074 | -0.215 | 0.129 | 0.047 | -0.351 | -0.027 | 0.100 | 0.557 | 0.075 | -0.067 |
| **POD** | 0.242 | -0.235 | 0.186 | -0.191 | 0.092 | 0.166 | 0.278 | 0.087 | 0.460 | -0.293 | -0.334 | -0.032 | 0.055 | 0.486 | 0.147 | -0.108 | -0.103 |
| **TPC** | 0.379 | 0.022 | -0.069 | 0.011 | 0.239 | 0.141 | -0.227 | -0.045 | 0.111 | -0.057 | 0.100 | 0.395 | 0.488 | -0.204 | 0.130 | 0.418 | -0.259 |
| **AsA** | 0.455 | 0.132 | 0.023 | 0.057 | -0.204 | 0.039 | -0.165 | 0.018 | 0.032 | -0.133 | 0.142 | 0.086 | -0.104 | 0.098 | 0.171 | 0.053 | 0.776 |
| **ESTR** | 0.032 | -0.107 | 0.234 | -0.286 | 0.058 | -0.409 | -0.206 | 0.566 | 0.264 | -0.101 | 0.338 | -0.237 | -0.074 | -0.228 | 0.051 | 0.054 | -0.067 |
| **PROT** | 0.255 | -0.082 | 0.126 | -0.111 | 0.064 | -0.015 | 0.625 | -0.169 | -0.210 | 0.056 | 0.305 | -0.410 | 0.257 | -0.082 | -0.170 | 0.238 | 0.097 |
| **AA** | -0.146 | 0.290 | -0.173 | -0.105 | -0.150 | 0.398 | -0.052 | -0.217 | 0.449 | 0.081 | 0.505 | -0.193 | -0.211 | 0.156 | 0.073 | 0.143 | -0.165 |
| **TOS** | 0.183 | 0.036 | 0.337 | 0.213 | -0.174 | -0.069 | 0.364 | 0.139 | 0.152 | 0.507 | 0.046 | 0.437 | -0.336 | -0.067 | 0.068 | 0.074 | -0.139 |
| **MDA** | -0.131 | 0.251 | -0.327 | -0.347 | 0.044 | 0.003 | 0.165 | 0.361 | -0.176 | 0.397 | -0.109 | -0.011 | 0.229 | 0.199 | 0.478 | 0.045 | 0.101 |
| **RS** | -0.121 | -0.304 | -0.169 | 0.420 | -0.322 | 0.187 | 0.062 | 0.377 | 0.078 | 0.017 | 0.226 | 0.018 | 0.377 | 0.096 | -0.109 | -0.144 | 0.036 |
| **TSS** | -0.296 | 0.257 | 0.410 | 0.046 | -0.101 | 0.256 | 0.044 | 0.198 | 0.009 | -0.102 | 0.063 | 0.116 | 0.348 | -0.009 | -0.134 | -0.096 | 0.121 |
| **NRS** | -0.191 | 0.405 | 0.462 | -0.205 | 0.099 | 0.117 | 0.003 | -0.046 | -0.038 | -0.100 | -0.077 | 0.092 | 0.087 | -0.064 | -0.055 | -0.001 | 0.086 |
| **TSP** | -0.331 | -0.274 | -0.062 | -0.049 | 0.125 | -0.042 | 0.236 | -0.282 | 0.357 | -0.032 | 0.035 | 0.148 | 0.099 | -0.483 | 0.372 | -0.184 | 0.300 |
| **ALB** | 0.028 | -0.047 | -0.127 | 0.100 | 0.662 | 0.432 | 0.027 | 0.318 | 0.067 | 0.161 | -0.038 | -0.046 | -0.244 | -0.130 | -0.289 | -0.015 | 0.224 |
| **GLOB** | -0.101 | -0.299 | -0.012 | -0.289 | -0.265 | 0.413 | 0.113 | 0.184 | -0.369 | -0.348 | 0.021 | 0.185 | -0.322 | -0.188 | 0.141 | 0.270 | -0.077 |
| **SSP** | -0.016 | -0.378 | 0.149 | -0.389 | 0.155 | 0.021 | -0.195 | -0.187 | -0.189 | 0.254 | 0.404 | 0.316 | 0.061 | 0.332 | -0.126 | -0.299 | 0.057 |

**SOD= superoxide dismutase; CAT= catalase; APX= ascorbate peroxidase; POD= peroxidase; TPC= total phenolic content; AsA= ascorbic acid; ESTR= esterase; PROT= protease; AA=Alpha amylase; TOS= total oxidant status; MDA= Malondialdehyde; RS= reducing sugars; TSS= total soluble sugars; NRS= non reducing sugars; TSP= total soluble protein; ALB= albumin; GLOB= globulin; SSP= salt soluble protein**
